# Intracellular Biosynthesis and Removal of Copper Nanoparticles by Dead Biomass of Yeast Isolated from the Wastewater of a Mine in the Brazilian Amazonia

**DOI:** 10.1371/journal.pone.0087968

**Published:** 2014-01-29

**Authors:** Marcia R. Salvadori, Rômulo A. Ando, Cláudio A. Oller do Nascimento, Benedito Corrêa

**Affiliations:** 1 Departamento de Microbiologia, Instituto de Ciências Biomédicas II, Universidade de São Paulo, São Paulo, São Paulo, Brazil; 2 Departamento de Química Fundamental, Instituto de Química, Universidade de São Paulo, São Paulo, São Paulo, Brazil; 3 Departamento de Engenharia Química, Politécnica, Universidade de São Paulo, São Paulo, São Paulo, Brazil; RMIT University, Australia

## Abstract

In this study was developed a natural process using a biological system for the biosynthesis of nanoparticles (NPs) and possible removal of copper from wastewater by dead biomass of the yeast *Rhodotorula mucilaginosa*. Dead and live biomass of *Rhodotorula mucilaginosa* was used to analyze the equilibrium and kinetics of copper biosorption by the yeast in function of the initial metal concentration, contact time, pH, temperature, agitation and inoculum volume. Dead biomass exhibited the highest biosorption capacity of copper, 26.2 mg g^−1^, which was achieved within 60 min of contact, at pH 5.0, temperature of 30°C, and agitation speed of 150 rpm. The equilibrium data were best described by the Langmuir isotherm and Kinetic analysis indicated a pseudo-second-order model. The average size, morphology and location of NPs biosynthesized by the yeast were determined by scanning electron microscopy (SEM), energy dispersive X-ray spectroscopy (EDS) and transmission electron microscopy (TEM). The shape of the intracellularly synthesized NPs was mainly spherical, with an average size of 10.5 nm. The X-ray photoelectron spectroscopy (XPS) analysis of the copper NPs confirmed the formation of metallic copper. The dead biomass of *Rhodotorula mucilaginosa* may be considered an efficiently bioprocess, being fast and low-cost to production of copper nanoparticles and also a probably nano-adsorbent of this metal ion in wastewater in bioremediation process.

## Introduction

The biosynthesis of NPs is viewed as a new fundamental building pillar of nanotechnology. Nanobiotechnology has revolutionized the production of nanomaterials which are environmentally safe products. Physico-chemical methods employ toxic chemicals and energy intensive routes, which make these choices eco-hazardous and preclude their use for biomedicine and clinical applications [1]. Therefore, environment friendly protocols need to be developed for the synthesis of nanomaterials. Copper NPs have potential industrial applications, including their use as wood preservatives, gas sensors, catalytic processes, high temperature superconductors and solar cells, among others [2], [3], [4]. The synthesis of different NPs by microorganisms such as prokaryotes (bacteria and actinomicetes) and eukaryotes (yeast, fungi and plant) has been reported in the literature [5]–[6]. Yeasts are preferred for the synthesis of nanomaterials due to their traditional use for bioleaching metals from mineral ores [7]–[8]. Wastewater from copper mining often contains high concentrations of this toxic metal produced during its extraction, beneficiation, and processing. Bioremediation of toxic metals such as copper through biosorption has received a great deal of attention in recent years not only as a scientific novelty, but also because of its potential industrial applications. This approach is competitive, effective, and cheap [9]. In this respect, studies have demonstrated the multi-metal tolerance of *Rhodotorula spp*, which may be of potential use for the treatment heavy metal-bearing wastewater [10]. Consequently, there has been considerable interest in developing methods for the biosynthesis of copper NPs as an alternative to physical and chemical methods. A literature review [11] revealed only few studies on the biosynthesis of copper NPs using fungi and none of the studies has used the yeast *Rhodotorula mucilaginosa* (*R. mucilaginosa*). On the other hand, several studies have investigated the biosynthesis of copper NPs using bacteria, for example, Hasan et al. [12], Ramanathan et al. [13], Singh et al. [14] among others. This work had the objective to enlarge the scope of biological systems for the biosynthesis of copper NPs and bioremediation. We explored for the first time the potential of the yeast *R. mucilaginosa*, for the removal and conversion of copper ions to copper NPs. Thus the goals of uptake and of a natural process to the production of copper NPs, have been achieved in the present study using dead biomass of *R. mucilaginosa*.

## Materials and Methods

### Ethics Statement

The company Vale S.A., owner of Sossego Mine, located in Canaã, Pará, in the Brazilian Amazon region, through the director of the Vale Technology Institute, Dr Luiz Eugenio Mello authorized the establishment and dissemination of the study featured in this research article, allowing the collection of material (water from pond of copper waste) supervised by company employees, whose material led to the isolation of the fungus under study. This field study did not involve manipulation of endangered or protected species by any government agency.

### Growth and maintenance of the organism

The yeast *R. mucilaginosa* was isolated from the water collected from a pond of copper waste from Sossego mine, located in Canãa dos Carajás, Pará, Brazilian Amazonia region (06°26′S latitude and 50°4′W longitude). The *R. mucilaginosa* was maintained and activated in YEPD agar medium (10 g yeast extract L^−1^, 20 g peptone L^−1^, 20 g glucose L^−1^ and 20 g agar L^−1^) and the media compounds were obtained from Oxoid (England) [15].

### Analysis of copper (II) tolerance

Copper tolerance of the isolated yeast was determined as the minimum inhibitory concentration (MIC) by the spot plate method [16]. For this purpose, YEPD agar medium plates containing different copper concentrations (50 to 3000 mg L^−1^) were prepared and inocula of the tested yeast were spotted onto the metal and control plates (plate without metal). The plates were incubated at 25°C for at least 5 days. The MIC was defined as the lowest concentration of the metal that inhibits visible growth of the isolate.

### Evaluation of copper NPs retention by the yeast

#### Preparation of the adsorbate solutions

All chemicals used in the present study were of analytical grade and were used without further purification. All dilutions were prepared in double-deionized water (Milli-Q Millipore 18.2 MΩcm^−1^ resistivity). The copper stock solution was prepared by dissolving CuCl_2_.2H_2_O (Carlo Erba, Italy) in double-deionized water. The working solutions were prepared by diluting this stock solution.

#### Biomass preparation

The yeast cells were grown in 500 mL Erlenmeyer flasks containing 100 mL YEPD broth (10 g yeast extract L^−1^, 20 g peptone L^−1^, 20 g glucose L^−1^). The flasks were incubated on a rotary shaker at 150 rpm for 20 h at 27°C. The biomass was harvested by centrifugation. Once harvested, the biomass was washed twice with double-deionized distilled water and was used directly for the experiment, corresponding to live biomass. For the production of dead biomass, an appropriate amount of live biomass was autoclaved.

#### Experimental design of the effects of physico-chemical factors on the efficiency of adsorption of copper NPs by the yeast

The effect of pH (2–6), temperature (20–60°C), contact time (5–360 min), initial copper concentration (25–600 mg L^−1^), and agitation rate (50–250 rpm) on the removal of copper was analyzed using analysis of variance models [17] with Bonferroni's multiple comparisons method for adjustment of p-values. These experiments were optimized at the desired pH, temperature, metal concentration, contact time, agitation rate and biosorbent dose (0.05–0.75 g) using 45 mL of a 100 mg L^−1^ of Cu (II) test solution in plastic flask.

Sorption experiments were carried out using several concentrations of copper (II) prepared by appropriate dilution of the copper (II) stock solution. The pH of the solutions was adjusted using HCl or NaOH aqueous solutions. The desired biomass dose was then added and the content of the flask was shaken for the desired contact time in a shaker at the required agitation rate. The reaction mixtures were filtered by vacuum filtration through a Millipore membrane. The filtrate was analyzed for metal concentrations by flame atomic absorption spectrophotometer (AAS). The efficiency (R) of metal removal was calculated using following equation:
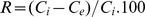
where C_i_ and C_e_ are initial and equilibrium metal concentrations, respectively. The metal uptake capacity, q_e_, was calculated using the following equation:
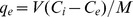
where q_e_ (mg g^−1^) is the biosorption capacity of the biosorbent at any time, M (g) is the biomass dose, and V (L) is the volume of the solution.

#### Sorption isotherms

The equilibrium data were fitted using the two most commonly adsorption models, Langmuir and Freundlich [18]. The biosorption was analyzed by the batch equilibrium technique using the following sorbent concentrations of 25–600 mg L^−1^. The linearized Langmuir isotherm model is:
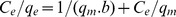
where q_m_ is the monolayer sorption capacity of the sorbent (mg g^−1^), and b is the Langmuir sorption constant (L mg ^−1^). The linearized Freundlich isotherm model is:
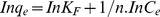
where K_F_ is a constant relating the biosorption capacity and 1/n is related to the adsorption intensity of adsorbent.

#### Biosorption kinetics

The experimental biosorption kinetic data were modeled using the pseudo-first-order, and pseudo-second-order models. The linear pseudo-first-order model [19] can be represented by the following equation:
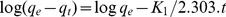
where, q_e_ (mg g^−1^) and q_t_ (mg g^−1^) are the amounts of adsorbed metal on the sorbent at the equilibrium time and at any time t, respectively, and K_1_ (min^−1^) is the rate constant of the pseudo-first-order adsorption process. The linear pseudo-second-order model [20] can be represented by the following equation:
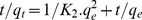
where K_2_ (g mg^−1^ min^−1^) is the equilibrium rate constant of pseudo-second-order.

### Intracellular biosynthesis of copper NPs by *R. mucilaginosa*

Only dead biomass of *R. mucilaginosa* was used for the analysis of copper NPs production since it exhibited high adsorption capacity of the copper metal ion than live biomass. The biosynthesis of copper NPs by dead biomass of *R. mucilaginosa* was investigated using the equilibrium data and a solution containing 100 mg L^−1^ copper (II). After reaction with the copper ions, sections of *R. mucilaginosa* cells were analyzed by transmission electron microscopy (TEM) (JEOL-1010) to determining the size, shape and location of copper NPs on the biosorbent. Analysis of small fragments of the biological material before and after the formation of copper NPs, were performed on pin stubs then coated with gold under vacuum, and examined by SEM (JEOL 6460 LV) equipped with an energy dispersive spectrometer (EDS) to identify the composition of elements of the sample. The XPS analysis was carried out at a pressure of less than 5×10^−7^ Pa using a commercial spectrometer (UNI-SPECS UHV System). The Mg Ká line was used (hν = 1253.6 eV) and the analyzer pass energy was set to 10 eV. The inelastic background of the C 1s, O 1s, N 1s and Cu 2p_3/2_ electron core-level spectra was subtracted using Shirley's method. The composition (at.%) of the near surface region was determined with an accuracy of ±10% from the ratio of the relative peak areas corrected by Scofield's sensitivity factors of the corresponding elements. The binding energy scale of the spectra was corrected using the C 1s hydrocarbon component of the fixed value of 285.0 eV. The spectra were fitted without placing constraints using multiple Voigt profiles. The width at half maximum (FWHM) varied between 1.2 and 2.1 eV and the accuracy of the peak positions was ±0.1 eV.

## Results and Discussion

The sensitivity towards at copper of the *R. mucilaginosa* when subjected to minimum inhibitory concentration at different metal concentrations (50–3000 mg L^−1^) showed that this yeast can survive within high level concentrations until 2000 mg L^−1^. The yeast uses several mechanisms to balance intracellular metal concentrations and counter metal toxicity. The resistance mechanism includes sequestration of heavy metals by metallothioneins through their high cysteine content and adsorption of heavy metal cations by the cellular walls [21]–[22].

### Effects of the physico-chemical factors on biosorption

**Figure 1:**
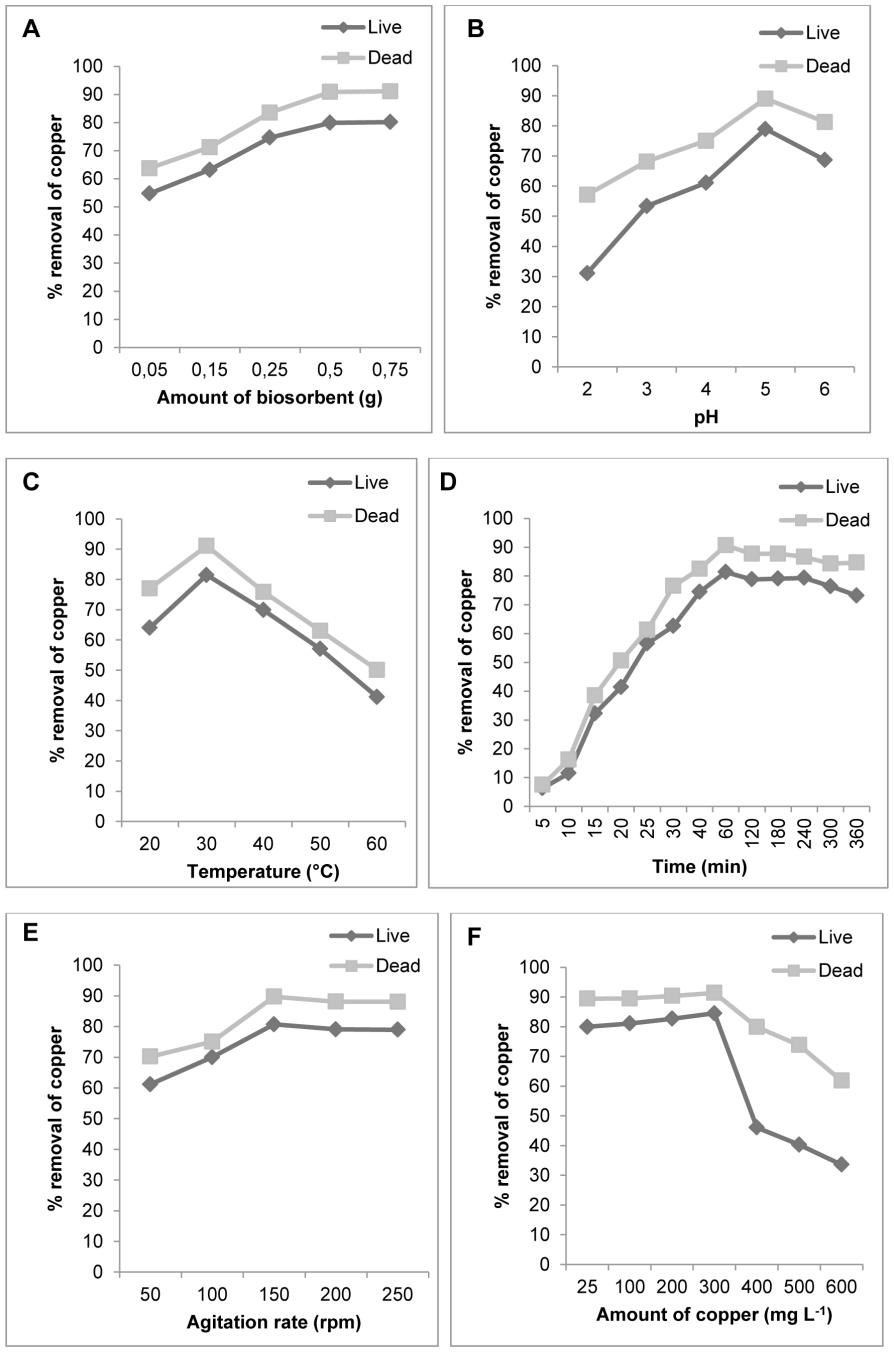
Sorption studies. Influence of the physico-chemical factors on the live and dead biomass of *R. mucilaginosa*. (A) Effect of the amount of biosorbent. (B) Effect of pH. (C) Effect of temperature. (D) Effect of contact time. (E) Effect of agitation rate. (F) Effect of initial copper concentration.

This study showed that copper removal by *R. mucilaginosa* biomass was significantly influenced by the effects and interactions with the physico-chemical factors. As can be seen in Figure 1, the percentage of copper removal was higher for dead biomass than live biomass for all parameters tested (p<0.0001 in all cases). Figure 1A shows that the dead biomass was more efficient in the removal of copper compared with the live biomass (p<0.0001 in the five levels of amount of biosorbent), indicating that dead biomass possess a higher affinity for copper than live biomass. The use of dead biomass for Cu (II) removal has the advantages that it is not toxic and, does not require growth media and nutrients for its maintenance [23]. Therefore the *R. mucilaginosa* may become a potential biosorbent in removing heavy metals from polluted water. The effectiveness of biomass concentration in percentage sorption of the metals was also observed to *Rhodotorula glutinis* [24]. In this study copper biosorption was maximum around pH 5.0, for the two types of biomass (Figure 1B, p<0.0001 in both cases), would be expected to interact more strongly with negatively charged binding sites on the biosorbent. At higher pH levels (pH 5), more ligands with negative charges would be exposed, with the subsequent increase in attraction sites to positively charged metal ion [25]. Some researchers have also investigated the effect of pH on the biosorption of toxic metals and found similar results [26], [27], [28], [29]. The maximum removal of copper was observed at 30°C for the two types of biomass (Figure 1C, p<0.0001 in both cases). The influence of temperature on the sorption of metals has also been reported for the yeast *Pichia stipitis* [28] and *Rhodotorula sp.* Y11 [29], for the bacterium *Morganella pyschrotolerans* [30] and for the plant *Cymbopogon flexuosus* [31] and others. The decrease in adsorption with increasing temperature may be due to the weakening of adsorptive forces between active sites of the adsorbents and the adsorbate species [32]. In Figure 1D, the graph shows sigmoidal kinetics for the types of biomass (p<0.0001 in both cases), which is characteristic of an enzyme-catalyzed reaction. The kinetics of copper NPs formation by dead biomass showed that more than 90% of the particles were formed within 60 min of reaction. The importance of contact time of the metal with the biomass has also been reported for *Rhodotorula sp.* Y11 [33]. The optimum copper removal was observed at an agitation speed of 150 rpm for both types of biomass (Figure 1E, p<0.0001 in both cases). At high agitation speeds, vortex phenomena occur and the suspension is no longer homogenous, a fact impairing metal removal [34]. The percentage of copper adsorption decreased with increasing metal concentration (50–500 mg L^−1^) for both types of biomass, as shown in Figure 1F (p<0.0001 in both cases). The same has been observed for fungi at concentration of Zn ranging from 100–400 mg L^−1^ [35], and for copper removal by *Rhodotorula mucilaginosa* RCL-11 and *Candida sp*. RCL-3 [36].

### Biosorption isotherms and adsorption kinetics models

**Figure 2:**
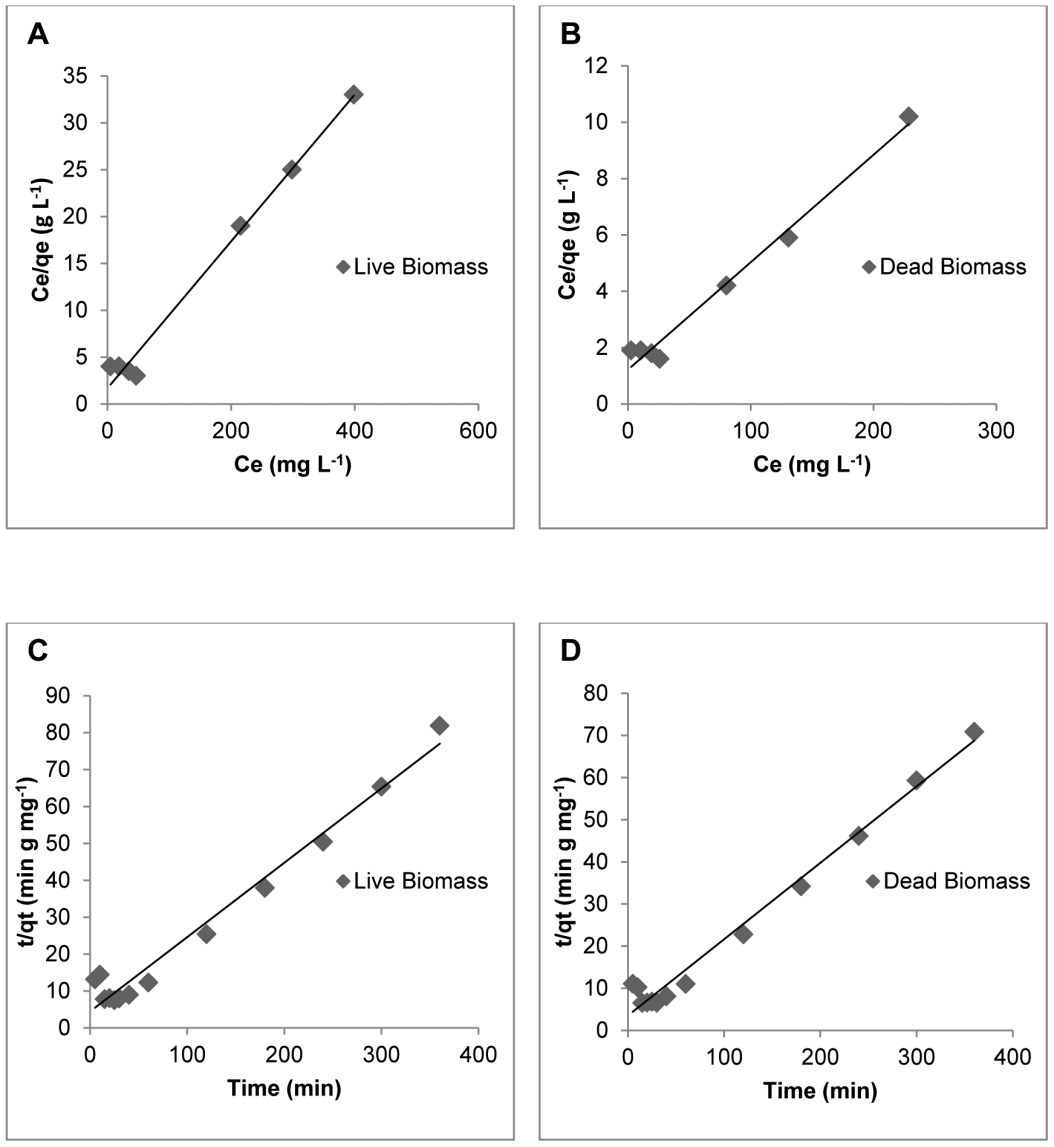
Equilibrium and kinetic data of the biosorption system. Langmuir plots for live (A) and dead (B) biomass. Pseudo second-order models for live (C) and dead (D) biomass.

Langmuir and Freundlich adsorption isotherms, were used to describe the adsorption data for a range of copper (II) concentrations (25–600 mg L^−1^). The Langmuir model better described the Cu (II) biosorption isotherms than the Freundlich model. The Langmuir isotherm for Cu (II) biosorption obtained of the two types of *R. mucilaginosa* biomass is shown in Figure 2A and Figure 2B. The isotherm constants, maximum loading capacity estimated by the Langmuir and Freundlich models, and regression coefficients are shown in Table 1. The maximum adsorption rate of Cu (II) by *R. mucilaginosa* (26.2 mg g^−1^) observed in this study was higher than the adsorption rates reported for other known biosorbents, such as *Pleurotus pulmonaris*, *Schizophyllum commune*, *Penicillium spp*, *Rhizopus arrhizus*, *Trichoderma viride*, *Pichia stiptis*, *Pycnoporus sanguineus*, with adsorption rates of 6.2, 1.52, 15.08, 19.0, 19.6, 15.85 and 2.76 mg g^−1^ respectively [37], [38], [39], [40], [28], [41]. Comparison with biosorbents of bacterial origin showed that the Cu (II) adsorption rate of *R. mucilaginosa* is comparable to that of *Bacillus subtilis* IAM 1026 (20.8 mg g^−1^) [42], but higher than the rates reported for the algae *Cladophora spp* and *Fucus vesiculosus* (14.28 and 23.4 mg g^−1^) [43]–[44].

**Table 1:** Adsorption isotherm parameters for Cu (II) ions with live and dead biomass of *R. mucilaginosa*.

	Langmuir model			Freundlich model		
Type of biomass	q_m_(mg g^−1^)	b (L mg^−1^)	*R^2^*	K_F_ (mg g^−1^)	1/n	*R^2^*
**Live**	12.7	0.046	0.988	0.59	0.44	0.641
**Dead**	26.2	0.031	0.984	0.74	0.61	0.850

Onto both types of biomasses of *R. mucilaginosa* the kinetics of copper biosorption were analysed using pseudo-first-order and pseudo-second-order models. All the constants and regression coefficients are shown in Table 2. In the present study, biosorption by *R. mucilaginosa* was best described using a pseudo-second-order kinetic model as shown in Figure 2C and Figure 2D. This adsorption kinetics is typical for the adsorption of divalent metals onto biosorbents [45].

**Table 2:** First and second-order adsorption rate constants.

	Pseudo-first-order		Pseudo-second-order	
Type of biomass	K_1_ (min^−1^)	*R^2^*	K_2_ (g mg^−1^ min^−1^)	R^2^
**Live**	7.36×10^−3^	0.474	9.45×10^−3^	0.972
**Dead**	6.90×10^−3^	0.502	9.69×10^−3^	0.981

### Synthesis of copper nanoparticles from dead biomass of *R. mucilaginosa*

**Figure 3:**
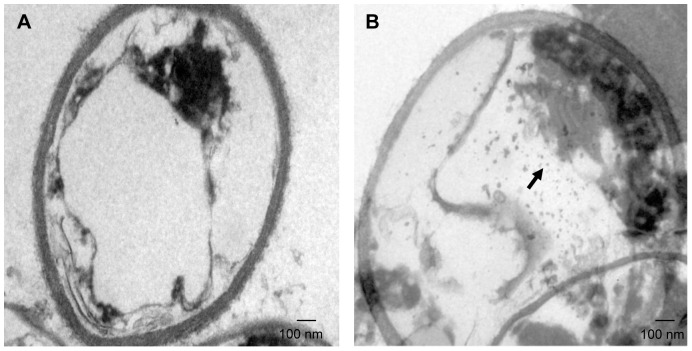
Transmission electron micrograph of *R. mucilaginosa* sections. (A) Control (without copper) and (B) Section of the yeast showing intracellular localization of copper NPs (arrow).

The researching of biosynthetic methods to the metals NPs formation is important in order to determine even more reliable and reproducible methods for its synthesis and have drawn attention as a simple and viable alternative to chemical procedures and physical methods. The information of the location of copper NPs in the yeast cell is important for elucidating the mechanism of their formation and was obtained through TEM analysis of thin sections of dead biomass (Figure 3). The results clearly showed the high concentration of intracellular copper NPs in the yeast cell, uniformly distributed (monodispersed) without significant agglomeration and was absent in control, the ultrastructural change such as shrinking of cytoplasmatic material was observed in control and in the biomass impregnated with copper due to autoclaving process. However, it was not observed the disruption of the cell wall likely due to the autoclaving method, whose principle consists in causing the death of the microorganisms by denaturation of some proteins [46]. It is important to note, that the cell wall of most yeasts, consists of about 85%–90% polysaccharide and 10%–15% protein and the polysaccharide component consists of a mixture of water-soluble mannan, alkali-soluble glucan, alkali insoluble glucan and small amounts of chitin [47], being these components of polysaccharides responsible for the high mechanical resistance of the cell wall [48] (Figure 3A and Figure 3B).

**Figure 4:**
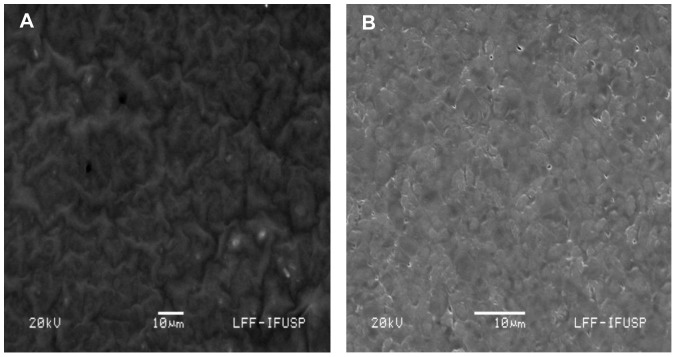
SEM-EDS analysis of the surface of dead biomass of *R. mucilaginosa*. (A) Before adsorption of copper ion and (B) after adsorption of copper ion.

**Figure 5:**
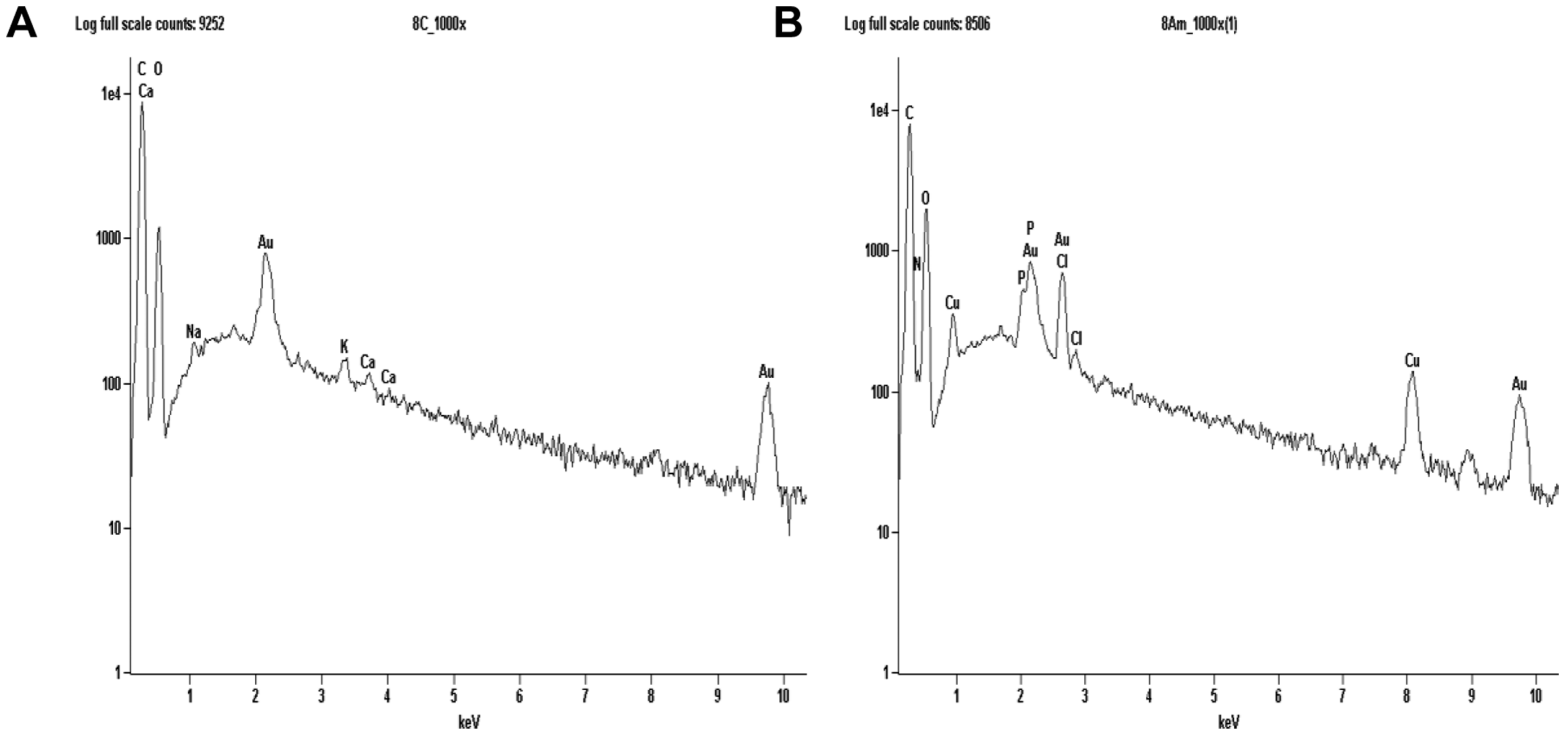
EDS spectra of dead biomass of *R. mucilaginosa*. (A) before exposure to copper solution and (B) after exposure to the metal confirming the presence of copper.

The two most important features that control the chemical, physical, optical and electronic properties of nanoscale materials are the size and shape of these particles [49]–[50]. As observed in Figure 3B, the majority of the particles are spherical in shape and with size of an average diameter of 10.5 nm. To confirm the presence of copper NPs in the dead biomass of yeast it was performed a spot profile SEM-EDS measurement. SEM micrographs recorded before and after biosorption of Cu (II) by yeast biomass was showed in Figure 4A and Figure 4B respectively. It was observed a surface modification by an increasing of the irregularity, after binding of copper NPs with the yeast biomass. The EDS spectrum recorded in the examined region of the yeast cells confirmed the presence of copper. (Figure 5A and Figure 5B). The signals for C, N, O and P may be originate from biomolecules that are bound to the surface of copper NPs.

**Figure 6:**
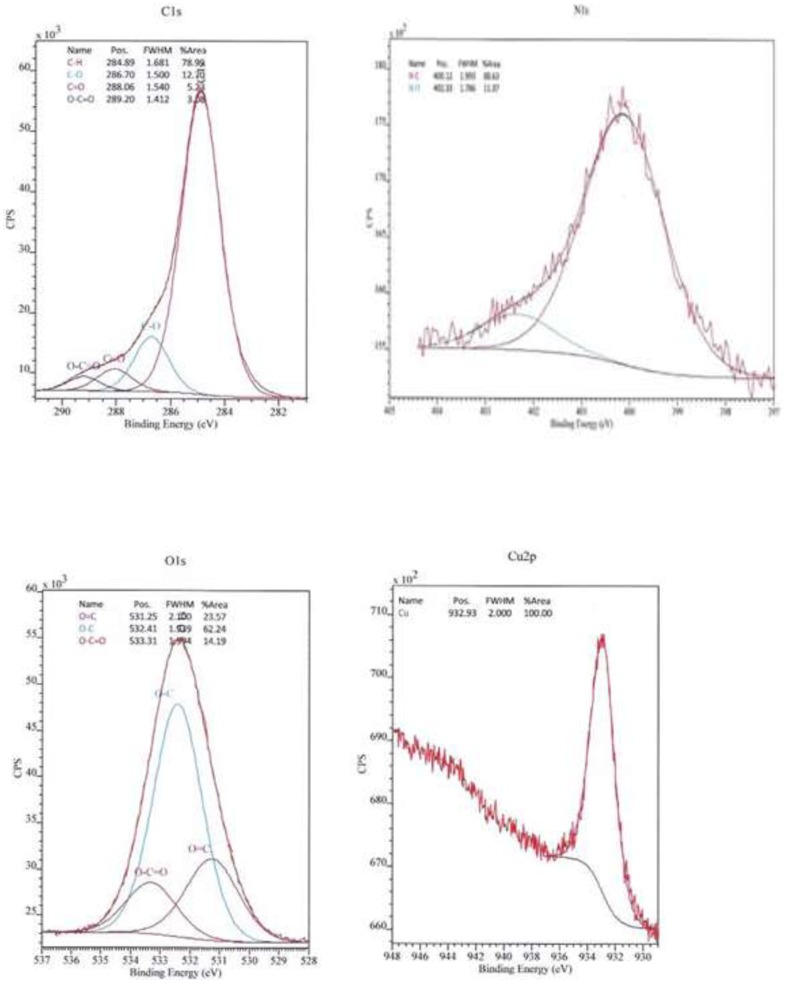
XPS spectra. C 1s, N 1s, O 1s and Cu 2p core level binding energies after biosynthesis of copper NPs.

Unfortunatly the intracellular mechanism formation of copper NPs by the dead biomass of yeast *R. mucilaginosa* is not fully understood at the moment. The ions copper possibly could diffuse through the cell wall and are reduced by enzymes present on the cytoplasmic membrane and within the cytoplasm. This enzyme-based pathway, also was proposed to silver nanoparticles synthesis [51]. The XPS spectra Figure 6 shows C 1s, N 1s, O 1s and Cu 2p core level after biosynthesis of copper NPs by dead biomass of *R. mucilaginosa*. As can be seen in the high resolution spectra of carbon (C 1s), the components of higher binding energy were deconvoluted within four elements. The main component at 284.8 eV is attributed to the hydrocarbon chains of the celular phase; the peak at 286.7 eV to the α- carbon, the peak at 288.0 eV to the carbonyl groups, and finally the peak at 289.2 eV to the carboxylic groups from the peptides/proteins bound to copper NPs [52]. The deconvoluted spectra of oxygen (O 1s), showed peaks at 531.2 eV, 532.4 eV and 533.3 eV related to the peaks found in the C 1s spectra. The spectra of nitrogen (N 1s) have two components, the main 400.1 eV and a lower at 402.3 eV. In the O 1s and N 1s spectra the major binding energies at 532.4 eV and 400.1 eV respectively were observed confirming the presence of proteins involving copper NPs, which suggests the possibility of these agents acting as capping agents [52]. The Cu 2p core level showed a sharp peak arise at 932,9 eV and it corresponds to the Cu 2p_3/2_ level characteristic of Cu(0) [53], [54], [55]. The presence of CuO (Cu (II)) phase can be excluded considering the lacking of the signal at 933.7 eV, as well as the presence of Cu_2_O, that can be is ruled out by the fact of no Cu 2p satellite peak appears with Cu_2_O [56]–[57].There are several reports in literature of yeast mediating the synthesis of nanoparticles of metal ions except to copper such as, peptide-bound CdS quantum crystallites by *Candida glubrata* [58], *Schizosaccharomyces pombe* also produced CdS nanoparticles [59], PbS nanocrystallites by *Torulopsis sp.* [60], gold nanoparticles by *Pichia jadinii* (*Candida utilis*) [61]–[62], the tropical marine yeast *Yarrowia lipolytica* NCIM 3589 also synthesized gold nanoparticles [63], Sb_2_O_3_ nanoparticles by *Saccharomyces cerevisiae* [64] and silver nanoparticles by yeast MKY3 [65]. Honary *et al*. [66] reported the production of copper nanoparticles by filamentous fungi, but the authors only used live biomass. The bioprocess proposed here, using dead biomass has the advantages, that it is not toxic, and does not require growth media and nutrients for its maintenance. The intracellular production of copper NPs by dead biomass of *R. mucilaginosa* was probably the result of a reduction process inside the cell mediated by proteins and enzymes present in the cytoplasm. However the type of proteins interacting with copper NPs remains to be determined and this knowledge would open new perspectives for a more efficient green synthesis of copper NPs.

### Conclusions

In summary, we described for the first time a biological economic template, and non-toxic using dead biomass of the red yeast *R. mucilaginosa*, that may be considered an efficient bioprocess to the synthesis of metallic copper NPs, and probable system for the adsorption of copper ions from wastewater. The dead biomass had a dual role, acting as a reducing agents and stabilizer during the formation of copper NPs, as well as in the uptake of copper ions during the bioremediation process. This natural method affords an amenable process to large scale commercial production. In future studies, we intend to characterize the role of biomacromolecules in the biosorption and bioreduction processes during the synthesis of copper NPs.

## References

[1] Singh AV, Patil R, Anand A, Milani P, Gade WN (2010) Biological synthesis of copper oxide nanopaticles using *Escherichia coli* . Curr Nanosci 6: 365–369.

[2] Evans P, Matsunaga H, Kiguchi M (2008) Large-scale application of nanotechnology for wood protection. Nat Nanotechnol 3: 577. 18838987 10.1038/nnano.2008.286

[3] Li Y, Liang J, Tao Z, Chen J (2007) CuO particles and plates: Synthesis and gas-sensor application. Mater Res Bull 43: 2380–2385.

[4] Guo Z, Liang X, Pereira T, Scaffaro R, Hahn HT (2007) CuO nanoparticle filled vinyl-ester resin nanocomposites: Fabrication, characterization and property analysis. Compos Sci Tech 67: 2036–2044.

[5] Bharde AA, Parikh RY, Baidakova M, Jouen S, Hannoyer B, et al (2008) Bacteria-mediated precursor-dependent biosynthesis of superparamagnetic iron oxide and iron sulfide nanoparticles. Langmuir 24: 5787–5794. 18454562 10.1021/la704019p

[6] Lang C, Schüler D, Faivre D (2007) Synthesis of magnetite nanoparticles for bio-and nanotechnology: genetic engineering and biomimetics of bacterial magnetosomes. Macromol Biosci 7: 144–151. 17295401 10.1002/mabi.200600235

[7] Kroger N, Deutzmann R, Sunper M (1999) Polycationic peptides from diatom biosilica that direct silica nanosphere formation. Science 286: 1129–1132. 10550045 10.1126/science.286.5442.1129

[8] He SY, Zhang Y, Guo Z, Gu N (2008) Biological synthesis of gold nanowires using extract of *Rhodopseudomonas capsulata* . Biotechnol Prog 24: 476–480. 18293997 10.1021/bp0703174

[9] Volesky B (2001) Detoxification of metal bearing effluents: biosorption for the next century. Hydrometallurgy 59: 203–216.

[10] Li Z, Yuan H, Hu X (2007) Cadmium-resistance in growing *Rhodotorula sp*. Y11. Bioresource Technol 99: 1339–1344. 10.1016/j.biortech.2007.02.004 17376676

[11] Varshney R, Bhadauria S, Gaur MS (2012) A review: Biological synthesis of silver and copper nanoparticles. Nano Biomed Eng 4: 99–106.

[12] Hasan SS, Sing S, Parikh RY, Dharne MS, Patole MS, et al (2008) Bacterial synthesis of copper/copper oxide nanoparticles. J Nanosci Nanotechnol 8: 3191–3196. 18681067 10.1166/jnn.2008.095

[13] Ramanathan R, Field MR, O'Mullane AP, Smooker PM, Bhargava SK, et al (2013) Aqueous phase synthesis of copper nanoparticles: a link between heavy metals resistance and nanoparticle synthesis ability in bacterial systems. Nanoscale 5: 2300–2306. 23223802 10.1039/c2nr32887a

[14] Singh V, Patil R, Ananda A, Milani P, Gade W (2010) Biological synthesis of copper oxide nanoparticles using *Escherichia coli* . Curr Nanosci 6: 365–369.

[15] Machado MD, Soares EV, Soares HMVM (2010) Removal of heavy metals using a brewer's yeast strain of *Saccharomyces cerevisiae*: Chemical Speciation as a tool in the prediction and improving of treatment efficiency of real electroplating effluents. J Hazard Mater 180: 347–353. 20452730 10.1016/j.jhazmat.2010.04.037

[16] Ahmad I, Ansari MI, Aqil F (2006) Biosorption of Ni, Cr and Cd by metal tolerante *Aspergillus niger* and *Penicillium sp* using single and multi-metal solution. Indian J Exp Biol 44: 73–76. 16430095

[17] Neter J, Kutner MK, Nachtsheim CJ, Wasserman W (1996) Applied linear statistical models. 4rd edn, Irwin: Chicago.

[18] Volesky B (2003) Biosorption process simulation tools. Hydrometallurgy 71: 179–190.

[19] Lagergren S (1898) About the theory of so called adsorption of soluble substances. Kung Sven Veten Hand 24: 1–39.

[20] Ho YS, Mckay G (1999) Pseudo-second-order model for sorption process. Process Biochem 34: 451–465.

[21] Gomes DS, Fragoso LC, Riger CJ (2002) Regulation of cadmium uptake by *Saccharomyces cerevisiae* . Biochem Biophys Acta 1573: 21–25. 12383937 10.1016/s0304-4165(02)00324-0

[22] Cho DH, Kim EY (2003) Characterization of Pb^2+^ biosorption from aqueous solution by *Rhodotorula glutinis* . Bioproc Biosyst Eng 25: 271–277. 10.1007/s00449-002-0315-8 14505170

[23] Bishnoi NR (2005) Garima (2005) Fungus – An alternative for bioremediation of heavy metal containing wastewater: A review. J Sci Ind Res 64: 93–100.

[24] Cho DH, Kim EY (2002) The mechanisms of Pb^2+^ removal from aqueous solution by *Rhodotorula glutinis* . Theories and Applications of Chem Eng 8: 4037–4040.

[25] Selatnia A, Boukazoula A, Kechid N, Bakti MZ, Chergui A, et al (2004) Biosorption of lead (II) from aqueous solution by a bacterial dead *Streptomyces rimosus* biomass. Biochem Eng J 19: 127–135.

[26] Fourest E, Roux JC (1992) Heavy metal biosorption by fungal micelial by-products: mechanisms and influence of pH. Appl Microbiol Biotechnol 37: 399–403.

[27] Göksungur Y, Üren S, Güvenc U (2005) Biosorption of cadmium and lead ions by ethanol treated waste baker's yeast biomass. Bioresource Technol 96: 103–109. 10.1016/j.biortech.2003.04.002 15364087

[28] Yilmazer P, Saracoglu N (2009) Bioaccumulation and biosorption of copper (II) and chromium (III) from aqueous solutions by *Pichia stiptis* yeast. J Chem Technol Biot 84: 604–610.

[29] Li Z, Yuan H (2006) Characterization of cadmium removal by *Rhodotorula sp.* Y11. Appl Microbiol Biotechnol 73: 458–463. 16736089 10.1007/s00253-006-0473-8

[30] Ramanthan R, O'Mullane AP, Parikh RY, Smooker PM, Bhargava SK, et al (2011) Bacterial kinetics-controlled shape-directed biosynthesis of silver nanoplates using *Morganella pyschrotolerans* . Langmuir 27: 714–719. 21142094 10.1021/la1036162

[31] Rai A, Singh A, Ahmad A, Sastry M (2006) Role of Halide ions and temperature on the morphology of biologically synthesized gold nanotriangles. Langmuir 22: 736–741. 16401125 10.1021/la052055q

[32] Pandey KK, Prasad G, Singh VN (1986) Use of wallastonite for the treatment of Cu (II) reach effluents. Water Air Soil Pollut 27: 287–296.

[33] Li Z, Yuan H, Hu X (2008) Cadmium-resistance in growing *Rhodotorula sp*. Y11. Bioresource Technol 99: 1339–1344. 10.1016/j.biortech.2007.02.004 17376676

[34] Liu YG, Fan T, Zeng GM, Li X, Tong Q, et al (2006) Removal of cadmium and zinc ions from aqueous solution by living *Aspergillus niger* . Trans Nonferrous Met Soc China 16: 681–686.

[35] Faryal R, Lodhi A, Hameed A (2006) Isolation, characterization and biosorption of zinc by indigenous fungal strains *Aspergillus fumigatus* RH05 and *Aspergillus flavus* RH07. Pak J Bot 38: 817–832.

[36] Villegas LB, Amoroso MJ, De Figueroa LIC (2005) Copper tolerant yeasts isolated from polluted area of Argentina. J Basic Microbiol 45: 381–391. 16187261 10.1002/jobm.200510569

[37] Veit MT, Tavares CRG, Gomes-da-Costa SM, Guedes TA (2005) Adsorption isotherms of copper (II) for two species of dead fungi biomasses. Process Biochem 40: 3303–3308.

[38] Du A, Cao L, Zhang R, Pan R (2009) Effects of a copper-resistant fungus on copper adsorption and chemical forms in soils. Water Air Soil Poll 201: 99–107.

[39] Rome L, Gadd DM (1987) Copper adsorption by *Rhizopus arrhizus*, *Cladosporium resinae* and *Penicillium italicum* . Appl Microbiol Biotechnol 26: 84–90.

[40] Kumar BN, Seshadri N, Ramana DKV, Seshaiah K, Reddy AVR (2011) Equilibrium, Thermodynamic and Kinetic studies on *Trichoderma viride* biomass as biosorbent for the removal of Cu (II) from water. Separ Sci Technol 46: 997–1004.

[41] Yahaya YA, Matdom M, Bhatia S (2008) Biosorption of copper (II) onto immobilized cells of *Pycnoporus sanguineus* from aqueous solution: Equilibrium and Kinetic studies. J Hazard Mater 161: 189–195. 18513859 10.1016/j.jhazmat.2008.03.104

[42] Nakajima A, Yasuda M, Yokoyama H, Ohya-Nishiguchi H, Kamada H (2001) Copper sorption by chemically treated *Micrococcus luteus* cells. World J Microb Biot 17: 343–347.

[43] Elmacy A, Yonar T, Özengin N (2007) Biosorption characteristics of copper (II), chromium (III), nickel (II) and lead (II) from aqueous solutions by *Chara sp* and *Cladophora sp* . Water Environ Res 79: 1000–1005. 17910369 10.2175/106143007x183961

[44] Grimm A, Zanzi R, Björnbom E, Cukierman AL (2008) Comparison of different types of biomasses of copper biosorption. Bioresource Technol 99: 2559–2565. 10.1016/j.biortech.2007.04.036 17570656

[45] Reddad Z, Gerent C, Andres Y, LeCloirec P (2002) Adsorption of several metal ions onto a low-cost biosorbents: kinetic and equilibrium studies. Environ Sci Technol 36: 2067–2073. 12026994 10.1021/es0102989

[46] Tortora GJ, Funke BR, Case CL (1998) Microbiology an Introduction. 6rd edn. California: Addison Wesley Longman.

[47] Nguyen TH, Fleet GH, Rogers PL (1998) Composition of the cell walls of several yeast species. Appl Microbiol Biotechnol 50: 206–212. 9763691 10.1007/s002530051278

[48] Nimrichter L, Rodrigues ML, Rodrigues EG, Travassos LR (2005) The multitude of targets for the immune system and drug therapy in the fungal cell wall. Microbes Infect 7: 789–798. 15823515 10.1016/j.micinf.2005.03.002

[49] Alivisatos AP (1996) Perspectives on the physical chemistry of semiconductor nanocrystals. J Phys Chem 100: 13226–13239.

[50] Aizpurua J, Hanarp P, Sutherland DS, Käll M, Bryant GW, et al (2003) Optical properties of gold nanorings. Phys Rev Lett 90: 57401–57404. 10.1103/PhysRevLett.90.057401 12633394

[51] Sanghi R, Verma P (2009) Biomimetic synthesis and characterization of protein capped silver nanoparticles. Bioresource Technol 100: 501–504. 10.1016/j.biortech.2008.05.048 18625550

[52] Bansal V, Ahamad A, Sastry M (2006) Fungus-mediated biotransformation of amorphous silica in rice husk to nanocrystalline Silica. J Am Chem Soc 128: 14059–14066. 17061888 10.1021/ja062113+

[53] Naumkin AV, Kraut-Vass A, Gaarenstroom SW, Powell CJ (2012) NIST X-ray Photoelectron Spectroscopy Database: NIST Standard Reference Database 20, v. 4.1. Available: http://www.srdata.nist.gov/XPS/. Accessed 03 December 2013.

[54] Briggs D, Seah MP (1990) Pratical Surface Analysis, Auger and X-ray Photoelectron Spectroscopy. Vol. 1. United Kingdom: John Wiley & Sons, Chichester.

[55] Jeong S, Woo K, Kim D, Lim S, Kim JS, et al (2008) Controlling the thickness of the surface oxide layer on Cu nanoparticles for the fabrication of conductive structures by ink-jet printing. Adv Funct Mater 18: 679–686.

[56] Zhang J, Wang Y, Cheng P, Yao YL (2006) Effect of pulsing parameters on laser ablative cleaning of copper oxides. J Appl Phys 99: 1–11.

[57] Ghodselahi T, Vesaghi MA, Shafielkhani A, Bachizadeh A, Lameii M (2008) XPS study of the Cu@Cu2O core-shell nanoparticle. Appl Surf Sci 255: 2730–2734.

[58] Dameron CT, Reese RN, Mehra RK, Kortan AR, Carroll PJ, et al (1989) Biosynthesis of cadmium sulphide quantum semiconductor crystallites. Nature 338: 596–597.

[59] Kowshik M, Vogel W, Urban J, Kulkarni SK, Paknikar KM (2002) Microbial Synthesis of Semiconductor PbS Nanocrystallites. Adv Mater 14: 815–818. 10.1002/bit.10233 12115128

[60] Kowshik M, Deshmukh N, Vogel W, Urban J, Kulkarni SK, et al (2002) Microbial synthesis of semiconductor CdS nanoparticles, their characterization, and their use in the fabrication of an ideal diode. Biotechnol Bioeng 78: 583–588. 12115128 10.1002/bit.10233

[61] Gericke M, Pinches A (2006) Biological synthesis of metal nanoparticles. Hydrometallurgy 83: 132–140.

[62] Gericke M, Pinches A (2006) Microbial production of gold nanoparticles. Gold Bull 39: 22–28.

[63] Agnihotri M, Joshi S, Kumar R, Zinjardes S (2009) Kulkarnis (2009) Biosynthesis of gold nanoparticles by the tropical marine yeast *Yarrowia lipolytica* NCIM3589. Mat Lett 63: 1231–1234.

[64] Jha AK, Prasad K, Prasad K (2009) A green low-cost biosynthesis of Sb_2_O_3_ nanoparticles. Biochem Eng J 43: 303–306. 10.1002/biot.200900144 19844916

[65] Kowshik M, Ashtaputer S, Kharrazi S, Vogel W, Urban J, et al (2003) Extracellular synthesis of silver nanoparticles by a silver-tolerant yeast strain MKY3. Nanotechnol 14: 95–100.

[66] Honary S, Barabadi H, Gharaei-Fathabad E, Naghib F (2012) Green synthesis of copper oxide nanoparticles using *Penicillium aurantiogriseum*, *Penicillium citrinum* and *Penicillium waksmanii* . Dig J Nanomater Bios 7: 999–1005.

